# TNF-*α* Induced the Enhanced Apoptosis of Mesenchymal Stem Cells in Ankylosing Spondylitis by Overexpressing TRAIL-R2

**DOI:** 10.1155/2017/4521324

**Published:** 2017-01-15

**Authors:** Zhenhua Liu, Liangbin Gao, Peng Wang, Zhongyu Xie, Shuizhong Cen, Yuxi Li, Xiaohua Wu, Le Wang, Hongjun Su, Wen Deng, Shan Wang, Deng Li, Jinteng Li, Yi Ouyang, Yanfeng Wu, Huiyong Shen

**Affiliations:** ^1^Department of Orthopedics, Sun Yat-sen Memorial Hospital, Sun Yat-sen University, 107 Yan Jiang Road West, Guangzhou 510120, China; ^2^Center for Biotherapy, Sun Yat-sen Memorial Hospital, Sun Yat-sen University, 107 Yan Jiang Road West, Guangzhou 510120, China; ^3^Institute of Orthopaedics and Traumatology, The Third Affiliated Hospital, Guangzhou Medical University, Guangzhou 510150, China

## Abstract

Ankylosing spondylitis (AS) is an autoimmune disease with unknown etiology. Dysregulated mesenchymal stem cells (MSCs) apoptosis may contribute to the pathogenesis of autoimmune diseases. However, apoptosis of MSCs from patients with AS (ASMSCs) has not been investigated yet. The present study aims to assess the apoptosis of bone marrow-derived ASMSCs and to investigate the underlying mechanisms of altered ASMSCs apoptosis. We successfully induced the apoptosis of ASMSCs and MSCs from healthy donors (HDMSCs) using the combination of tumor necrosis factor alpha (TNF-*α*) and cycloheximide (CHX). We found that ASMSCs treated with TNF-*α* and CHX showed higher apoptosis levels compared to HDMSCs. During apoptosis, ASMSCs expressed significantly more TRAIL-R2, which activated both the death receptor pathway and mitochondria pathway by increasing the expression of FADD, cleaved caspase-8, cytosolic cytochrome C, and cleaved caspase-3. Inhibiting TRAIL-R2 expression using shRNA eliminated the apoptosis differences between HDMSCs and ASMSCs by partially reducing ASMSCs apoptosis but minimally affecting that of HDMSCs. Furthermore, the expression of FADD, cleaved caspase-8, cytosolic cytochrome C, and cleaved caspase-3 were comparable between HDMSCs and ASMSCs after TRAIL-R2 inhibition. These results indicated that increased TRAIL-R2 expression results in enhanced ASMSCs apoptosis and may contribute to AS pathogenesis.

## 1. Introduction

Ankylosing spondylitis (AS) is an agnogenic autoimmune disease characterized by two central pathological changes: chronic inflammation and pathological osteogenesis [1]. Recent studies have focused on the pathogenesis of AS, and several risk factors have been reported to be significantly correlated with AS, including human leukocyte antigen B27 (HLA-B27) genetic susceptibility [2], gene polymorphisms [3], environmental triggers [4–6], and mechanical stress [7]. However, none of these risk factors are solely responsible for the pathogenesis of AS.

Mesenchymal stem cells (MSCs) are a heterogeneous population of cells that can be isolated from many tissues, including bone marrow. MSCs play a vital role in health and disease for their immunomodulation potentials, self-renewal, and multipotent differentiation capacities [8, 9]. Recent studies have demonstrated that MSCs dysfunction contributes to many diseases [10, 11]. Consistent with these reports, our previous study observed an increased osteogenic differentiation capacity of MSCs from AS patients (ASMSCs) compared with MSCs from healthy donors (HDMSCs) [12], which may partly account for the pathological osteogenesis in AS. However, it remains largely unknown whether ASMSCs are involved in the chronic inflammation of AS.

Tumor necrosis factor alpha (TNF-*α*) is a pleiotropic cytokine involved in a variety of physiological and pathological processes ranging from inflammatory cytokine production to cell survival and apoptosis [13]. Due to its ability to stimulate inflammation, overproduction of TNF-*α* has been suggested to contribute to the pathogenesis of AS [14]. This hypothesis has been confirmed by the therapeutic effect observed with TNF-*α* blockers in AS treatment [15, 16]. In addition to triggering a proinflammatory response, TNF-*α* also can induce either cell survival or apoptosis. Protein inhibitors (such as cycloheximide, CHX) can direct TNF-*α* to induce the apoptosis of mammalian cells [17]. Apoptosis is a normal physiological cell suicide process that plays a crucial role in homeostasis. A large quantity of evidence supports the idea that the defective apoptosis of immune cells can lead to autoimmune diseases [18]. Furthermore, a recent study indicated that enhanced MSCs apoptosis may also contribute to the pathogenesis of autoimmune diseases [19]. Thus, studying the apoptosis of MSCs may improve our understanding of the pathogenesis of autoimmune diseases, including AS. However, TNF-*α*-induced apoptosis of ASMSCs has not been investigated yet.

TNF-related apoptosis-inducing ligand receptor 2 (TRAIL-R2) is a specific cell surface receptor that belongs to the TNF receptor superfamily [20]. TRAIL-R2 is expressed in many cell types, including MSCs [21, 22]. Upon binding with its specific ligand, TRAIL, TRAIL-R2 initiates the recruitment of Fas-associated protein with death domain (FADD) and signaling molecules (such as caspase-8) to form the death-inducing signaling complex (DISC). It then activates downstream caspases (such as caspase-3) and leads to apoptosis [23].

In this study, we investigated the apoptosis of ASMSCs and a possible underlying mechanism. Our results showed that ASMSCs were more susceptible to TNF-*α*/CHX-induced apoptosis than HDMSCs due to increased TRAIL-R2 expression. We speculate that the increased expression of TRAIL-R2 results in the enhanced apoptosis of ASMSCs, which may contribute to AS pathogenesis.

## 2. Materials and Methods

### 2.1. Recruitment of AS Patients and Healthy Donors

This study was approved by the ethics committee of Sun Yat-sen Memorial Hospital, Sun Yat-sen University, Guangzhou, China. There were 28 healthy donors and 22 patients with AS (diagnosed according to the New York modified criteria [24]) who agreed to participate in this study (detailed characteristics of the participants are presented in supplemental Table 1 in Supplementary Material available online at https://doi.org/10.1155/2017/4521324). Written informed consent was obtained from each participant. To minimize the impact of therapy, all patients discontinued any treatment for 2 weeks before bone marrow puncture.

### 2.2. MSCs Isolation, Expansion, and Identification

MSCs were isolated from the iliac crest bone marrow of all the study subjects and purified using density gradient centrifugation as previously described [25]. After being resuspended in Dulbecco's Modified Eagle's Medium (DMEM, Gibco, USA) containing 10% heat-inactivated fetal bovine serum (FBS, Sijiqing, China), MSCs were seeded in 25 cm^2^ flasks and cultured in an incubator with 5% CO_2_ at 37°C. To remove the suspended cells, the medium was replaced 48 hours later and every 3 days thereafter. At 80–90% confluence, the adherent cells were recovered using 0.25% Trypsin containing 0.53 mM EDTA (Gibco, USA) and replated in 75 cm^2^ flasks as passage 1. MSCs were expanded and used at passages 4 for all experiments. Surface markers of MSCs were detected using flow cytometry (FCM), which was performed on BD Influx cell sorter (BD, USA). CD14-APC, CD29-PE, CD44-FITC, CD45-APC, CD105-FITC, and HLA DR-PerCP antibodies were used (all from BD, USA).

### 2.3. Cell Proliferation Assay

MSCs at the same passage were seeded in 96-well plates in DMEM with 10% FBS. Medium without cells served as negative control. The proliferation ability of MSCs was detected using Cell Counting Kit-8 (Dojindo, Japan) following the manufacturer's instructions.

### 2.4. Apoptosis Induction

MSCs were separately seeded in 6-well plates at a density of 1 × 10^5^ cells/well in DMEM with 10% FBS. After 24 hours, the medium was removed, and MSCs were washed three times with phosphate-buffered saline (PBS). The same sample of MSCs was separately cultured for 6 hours with four different types of media: fresh DMEM with 10% FBS (control group), fresh DMEM with 10% FBS and 20 ng/mL TNF-*α* (TNF-*α* group), fresh DMEM with 10% FBS and 10 *μ*g/mL CHX (CHX group), and fresh DMEM with 10% FBS, 20 ng/mL TNF-*α*, and 10 *μ*g/mL CHX (TNF-*α*/CHX group). After 6 hours, MSCs were harvested for the experiments.

### 2.5. PE-Annexin V and FCM for the Detection of MSC Apoptosis

Apoptotic cells were stained using PE-Annexin V Apoptosis Detection Kit I (BD, USA) according to the manufacturer's protocol. First, the cells were washed with PBS and resuspended in binding buffer. Second, 100 *μ*L of the solution was transferred to a 5 mL culture tube. Third, 5 *μ*L of PE-Annexin V and 5 *μ*L of 7-AAD were added to the cells. The mixture was gently vortexed and incubated for 15 minutes at room temperature in the dark. Then, 400 *μ*L of binding buffer was added to each tube and FCM analysis of apoptosis was performed immediately. Data were analyzed using FlowJo software.

### 2.6. Proteome Profiler Antibody Array Analysis

The expression of apoptosis-related proteins in MSCs was detected using the Proteome Profiler™ Human Apoptosis Array Kit (ARY099, R&D, USA) according to the manufacturer's instructions. Briefly, equal amounts of protein from HDMSCs and ASMSCs were incubated with the membranes overnight at 4°C. Then the membranes were washed and incubated with reconstituted Detection Antibody Cocktail for 1 hour. After that, the membranes were washed again and incubated with Streptavidin-HRP for 30 minutes. Finally, the protein expressions were detected using Chemi Reagent Mix. The intensity of the arrays was analyzed using VisionWorks® LS Image Acquisition and Analysis Software (Analytik Jena AG, German). The intensity of the repeat spots was averaged and then standardized using the reference spots.

### 2.7. Quantitative Real-Time Polymerase Chain Reaction (qRT-PCR)

Total RNA of MSCs was extracted using TRIzol reagent (Invitrogen, USA) and transcribed into cDNA using PrimeScript™ RT Master Mix (Takara, Japan). qRT-PCR was carried out in triplicate for each sample on a LightCycler® 480 PCR System (Roche, Switzerland) using SYBR® Premix Ex Taq™ (Takara, Japan) as previously described [26]. GAPDH was used as the housekeeping gene. Primers of related genes are shown in supplemental Table 2. The data were standardized based on GAPDH expression, and the 2^−ΔΔCt^ method was used to analyze the relative expression of each gene.

### 2.8. Western Blot

MSCs were lysed and proteins were quantified as described [13]. Mitochondrial and cytoplasmic proteins from MSCs were isolated using the Cell Mitochondria Isolation Kit (Beyotime, China) as described [27]. After boiling with sample loading buffer (Beyotime, China), equal amounts of protein extracts were separated and transferred to polyvinylidene fluoride (PVDF) membranes (Millipore, Germany). The membranes were blocked and incubated overnight at 4°C with primary antibodies against *β*-actin, pro-caspase-3/cleaved caspase-3, FADD, TRAIL-R2, cleaved caspase-8, TNFR1, Fas (all from CST, USA), and cytochrome C (R&D, USA). After washing three times, the membranes were incubated for 1 hour with horseradish peroxidase- (HRP-) conjugated secondary antibody (Santa Cruz, USA). Immobilon Western Chemiluminescent HRP Substrate (Millipore, Germany) was used to detect the antibody-antigen complexes.

### 2.9. Lentivirus Construction and Infection

Lentiviruses encoding short hairpin RNA (shRNA) for TRAIL-R2 were constructed with a target sequence of 5′-GCAAATATGGACAGGACTATA-3′ (Lv-TRAIL-R2). The sequence for the negative control shRNA was 5′-TTCTCCGAACGTGTCACGTTTC-3′ (Lv-NC). Lentiviruses were generated by cotransfecting pGLVH1/GFP/Puro (Gene Pharma, China) and packaging plasmids (pGag/Pol, pRev, and pVSV-G) into 293T cells. Culture supernatants containing lentiviruses were filtered and concentrated 72 hours after transfection. Lentiviruses (1 × 10^9^ TU/mL) and Polybrene (5 *μ*g/mL) were added to the medium and incubated with the MSCs for 24 hours at a multiplicity of infection (MOI) of 50. The related experiments were conducted after 6 hours of apoptosis induction.

### 2.10. Statistical Analyses

Experiments and analyses were separately completed by the different authors listed in this study. Statistical analysis was performed using SPSS 22.0 software (Chicago, USA). The data are expressed as the mean ± standard deviation (SD). The differences between two groups were determined by Student's* t*-test, and the differences among three or more groups were determined by ANOVA.* P* values < 0.05 were considered significant.

## 3. Results

### 3.1. HDMSCs and ASMSCs Had the Same Morphology, Phenotype, and Proliferation Rate

**Figure 1:**
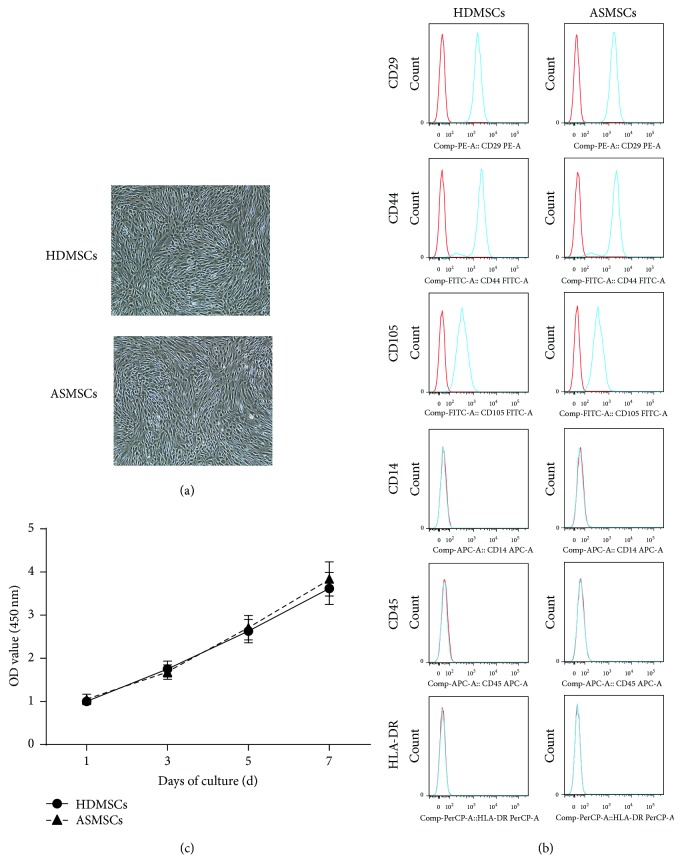
HDMSCs and ASMSCs shared the same morphology, phenotype, and proliferation capacity. (a) Both HDMSCs and ASMSCs were plastic-adherent and spindle-shaped. (b) Both HDMSCs (*n* = 28) and ASMSCs (*n* = 22) were positive for CD29, CD44, and CD105 and negative for CD14, CD45, and HLA-DR, suggesting a typical MSC phenotype. (c) The proliferation capacities of HDMSCs (*n* = 28) and ASMSCs (*n* = 22) were similar when cultured in DMEM with 10% FBS from 1 to 7 days. Optical density (OD) values are presented as the mean ± SD.

As typical MSCs, both HDMSCs and ASMSCs were plastic-adherent, spindle-shaped cells (Figure 1(a)) that constitutively expressed CD29, CD44, and CD105 but not CD14, CD45, or HLA-DR (Figure 1(b)). HDMSCs and ASMSCs proliferated similarly in DMEM with 10% FBS for 1–7 days (Figure 1(c)). No differences were found between HDMSCs and ASMSCs in morphology, phenotype, or proliferation rate.

### 3.2. ASMSCs Showed Higher Levels of Apoptosis Compared to HDMSCs after Treatment with TNF-*α*/CHX

**Figure 2:**
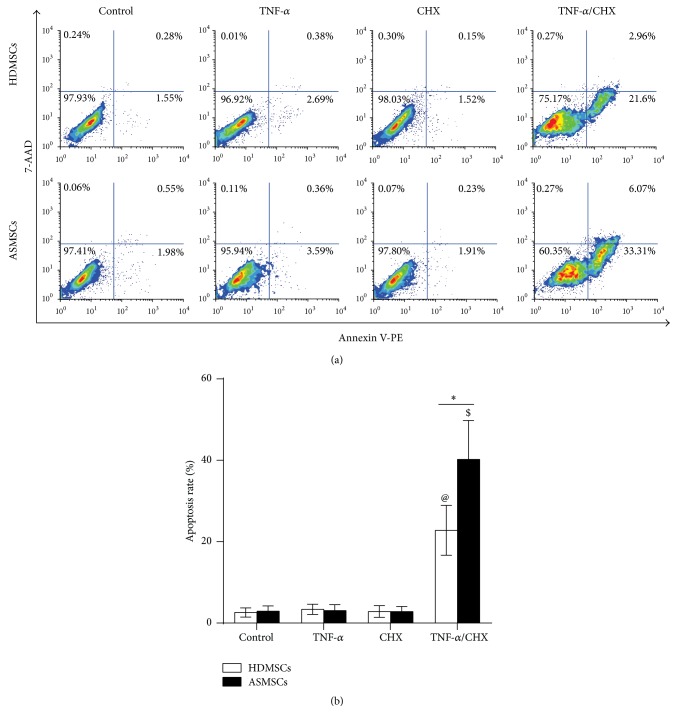
ASMSCs were more susceptible to TNF-*α*/CHX-induced apoptosis compared to HDMSCs. (a) Apoptosis of HDMSCs (*n* = 28) and ASMSCs (*n* = 22) was determined using FCM. Cells in the upper-right and lower-right quadrants represent apoptotic cells; the apoptotic rate was calculated in (b). After a 6-hour culture, only treatment with TNF-*α*/CHX induced significant MSC apoptosis. Treatment with control medium or with TNF-*α* or CHX alone did not induce significant apoptosis. ASMSCs had a higher rate of apoptosis than HDMSCs in the TNF-*α*/CHX treatment group. Values are presented as the mean ± SD. *∗* indicates *P* < 0.05 between HDMSCs and ASMSCs. @ indicates *P* < 0.05 for the control group compared with the other HDMSC groups. $ indicates *P* < 0.05 for the control group compared with the other ASMSC groups.

Apoptotic cells were stained with phycoerythrin- (PE-) Annexin V/7-amino-actinomycin (7-AAD) and identified by flow cytometry (FCM). Negative Annexin V and 7-AAD staining indicated cells that were not undergoing apoptosis. Annexin V-positive and 7-AAD-negative cells were considered in the early stage apoptosis; Annexin V- and 7-AAD-positive cells were in late-stage apoptosis or already dead.  Figure 2 shows that treatment with TNF-*α*/CHX caused significant apoptosis of HDMSCs and ASMSCs and that the apoptosis rate of ASMSCs was nearly twice as high as that of HDMSCs. TNF-*α* or CHX treatment alone did not induce significant apoptosis within 6 hours, similar to the control group, and no difference between HDMSCs and ASMSCs was found in these three groups. These results demonstrated that ASMSCs were more susceptible to apoptosis compared to HDMSCs after treatment with TNF-*α*/CHX. Considering that TNF-*α* or CHX alone cannot induce significant apoptosis of MSCs in 6 hours, only the results from the TNF-*α*/CHX group and the control group are reported for the following experiments.

### 3.3. ASMSCs Showed Increased Protein Expression of Cleaved Caspase-3, TRAIL-R2, and FADD and Increased Gene Expression of TRAIL-R2 and FADD during Apoptosis

**Figure 3:**
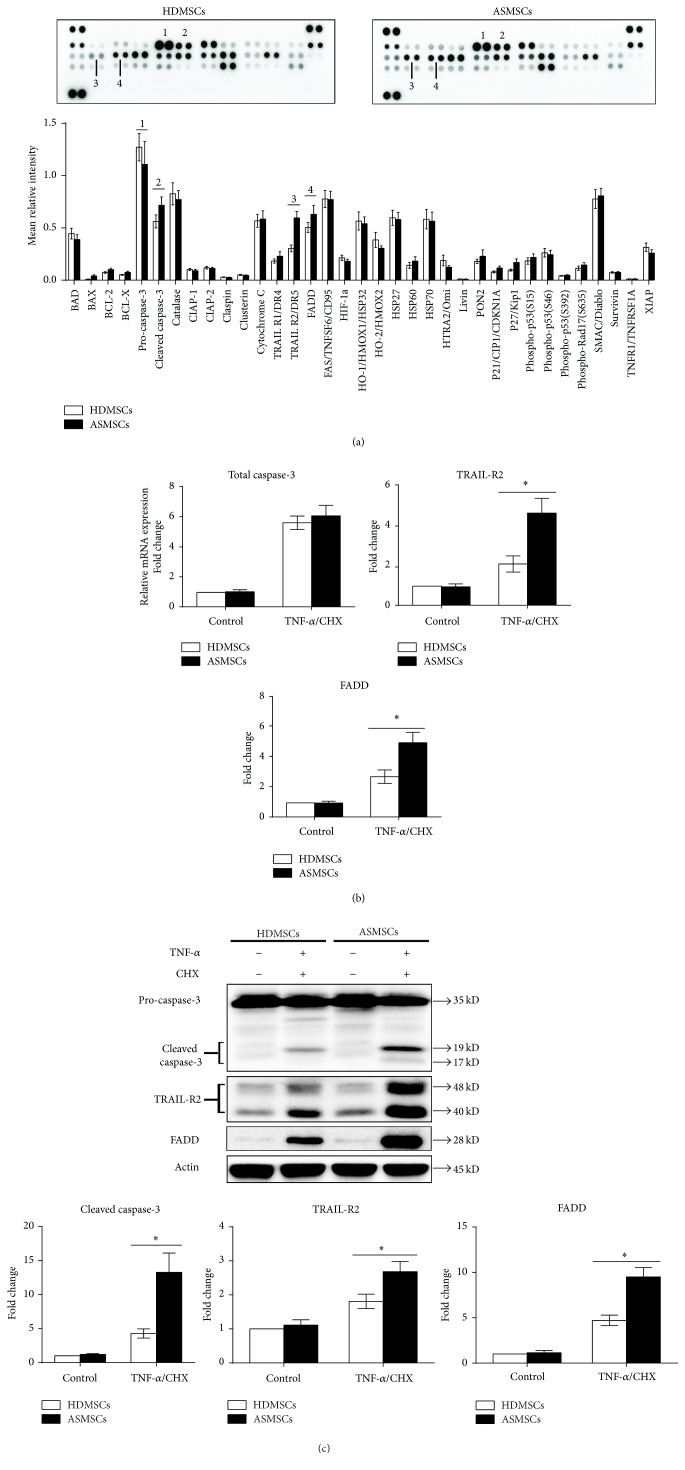
ASMSCs had increased protein expression of cleaved caspase-3, TRAIL-R2, and FADD and increased gene expression of TRAIL-R2 and FADD during apoptosis. (a) The Proteome Profiler™ Human Apoptosis Array Kit was used to determine the expression levels of 35 apoptosis-related proteins in HDMSCs (*n* = 6) and ASMSCs (*n* = 6) after treatment with TNF-*α*/CHX. The intensity of each pair of spots represents repeated measures of one protein. ASMSCs had lower pro-caspase-3 (a.1) expression but higher cleaved caspase-3 (a.2), TRAIL-R2 (a.3), and FADD (a.4) expression levels than HDMSCs. No significant differences in the expression of the remaining 31 proteins were found between HDMSCs and ASMSCs (b) qRT-PCR was performed to confirm the results of the Proteome Profiler™ Human Apoptosis Array. The results of TRAIL-R2 and FADD in HDMSCs (*n* = 28) and ASMSCs (*n* = 22) were consistent with those of the Proteome Profiler™ Human Apoptosis Array. However, total caspase-3 mRNA expression was not different between HDMSCs and ASMSCs. (c) Western blot analysis was performed on proteins isolated from HDMSCs (*n* = 28) and ASMSCs (*n* = 22) to further confirm the results of the Proteome Profiler™ Human Apoptosis Array, and the results of both methods were in agreement. Values are presented as the mean ± SD. *∗* indicates *P* < 0.05 between HDMSCs and ASMSCs.

To explore the mechanism involved in the differences in apoptosis between HDMSCs and ASMSCs, 35 apoptosis-related proteins were detected using the Proteome Profiler™ Human Apoptosis Array Kit. The results showed that ASMSCs had higher cleaved caspase-3 expression but lower pro-caspase-3 expression than HDMSCs after treatment with TNF-*α*/CHX, which further confirmed the increased apoptosis of ASMSCs. Additionally, significantly higher expression levels of TRAIL-R2 and FADD were also found in ASMSCs than HDMSCs. No significant differences in the expression levels of the remaining 31 proteins were found between HDMSCs and ASMSCs (Figure 3(a)). qRT-PCR and western blotting assays were performed to confirm the results of the Proteome Profiler™ Human Apoptosis Array analysis. The mRNA expression levels of TRAIL-R2 and FADD were consistent with the results of the Proteome Profiler™ Human Apoptosis Array analysis, but the mRNA expression levels of total caspase-3 were not. No significant difference in total caspase-3 mRNA expression was found between HDMSCs and ASMSCs either with or without TNF-*α*/CHX treatment (Figure 3(b)). The protein expression levels of cleaved caspase-3, pro-caspase-3, TRAIL-R2, and FADD were consistent with the results of the Proteome Profiler™ Human Apoptosis Array analysis (Figure 3(c)). Because caspase-3 initiates apoptosis through its cleaved form, the comparable total caspase-3 mRNA levels between HDMSCs and ASMSCs may suggest that ASMSCs were more susceptible to apoptosis compared to HDMSCs via an increase in the cleavage of caspase-3 rather than an increase in the mRNA expression of caspase-3.

### 3.4. The Death Receptor and Mitochondrial Pathways Were Both Involved in the Apoptosis of MSCs

**Figure 4:**
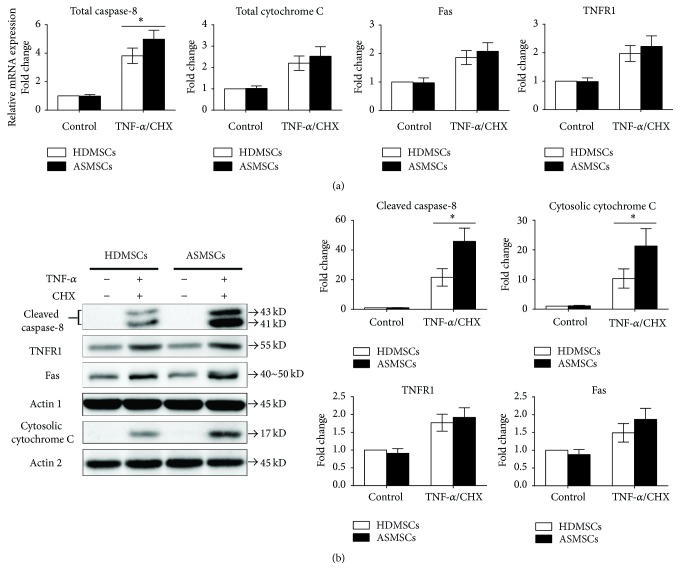
The DR and mitochondrial pathways were both involved in MSC apoptosis. (a) qRT-PCR was performed to compare the gene expressions of total caspase-8, total cytochrome C, Fas, and TNFR1 between HDMSCs (*n* = 28) and ASMSCs (*n* = 22). ASMSCs expressed much higher gene expression levels of total caspase-8 than HDMSCs after treatment with TNF-*α*/CHX, while no difference was found between HDMSCs and ASMSCs in total cytochrome C, Fas, or TNFR1 gene expression levels. (b) A western blot was performed to compare the protein expressions of cleaved caspase-8, cytosolic cytochrome C, TNFR1, and Fas between HDMSCs (*n* = 28) and ASMSCs (*n* = 22). After treatment with TNF-*α*/CHX, ASMSCs had higher cleaved caspase-8 and cytosolic cytochrome C protein expression levels than HDMSCs and similar protein levels of TNFR1 and Fas as HDMSCs. Fold changes are presented as the mean ± SD. *∗* indicates *P* < 0.05 between HDMSCs and ASMSCs.

Apoptosis proceeds mainly by the death receptor (DR) pathway and the mitochondrial pathway. The cleavage of caspase-8 and the release of cytochrome C from the mitochondria to the cytoplasm, respectively, represent the activation of the DR pathway and mitochondrial pathway. To determine whether these two pathways were involved in the altered apoptosis of ASMSCs, we measured the mRNA expression levels of total caspase-8 and total cytochrome C, as well as the active forms of the corresponding proteins: cleaved caspase-8 and cytosolic cytochrome C. Our results showed that the mRNA expression of both total caspase-8 and cytochrome C increased after treatment with TNF-*α*/CHX. ASMSCs expressed significantly higher mRNA level of total caspase-8 than HDMSCs, while no difference was found between HDMSCs and ASMSCs in total cytochrome C (Figure 4(a)). The protein expression of both cytosolic cytochrome C and cleaved caspase-8 increased after treatment with TNF-*α*/CHX, and compared with HDMSCs, ASMSCs exhibited much higher expression of these two proteins (Figure 4(b)). Because cytochrome C needs to be released into the cytoplasm for apoptosis to occur, the mRNA expression level of total cytochrome C was not as meaningful as the cytosolic protein expression. We also detected the gene and protein expression of TNFR1 and Fas, two important receptors that, in addition to TRAIL-R2, interact with FADD. However, although both the gene and protein expression levels increased after treatment with TNF-*α*/CHX, no difference in gene or protein expression was found between the two groups, either with or without TNF-*α*/CHX treatment (Figures 4(a) and 4(b)). These results together suggest that both the DR pathway and the mitochondrial pathway are involved in the altered apoptosis of ASMSCs; neither TNFR1 nor Fas accounted for the increased expression of FADD in the DR pathway.

### 3.5. Lentiviruses Encoding shRNA-TRAIL-R2 Partly Inhibited the Apoptosis of ASMSCs but Had a Limited Effect on the Apoptosis of HDMSCs

**Figure 5:**
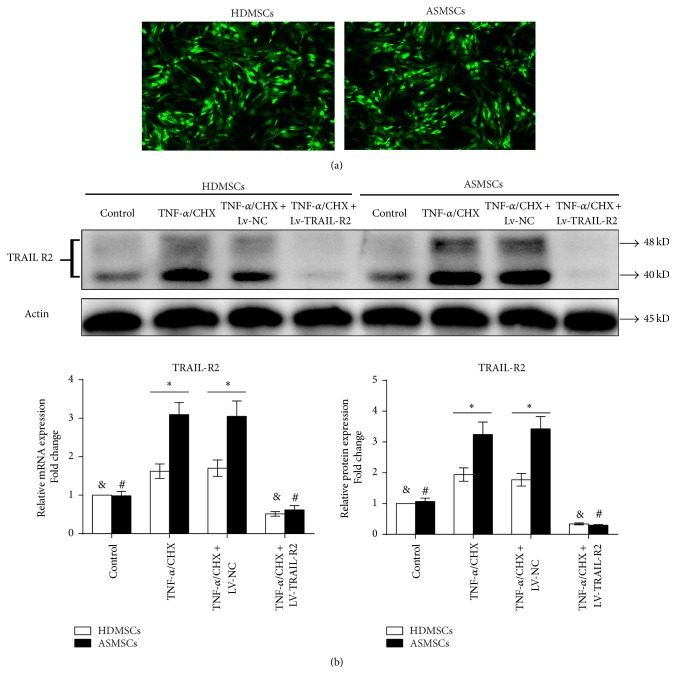
The transduction efficiencies of lentiviruses encoding shRNA-TRAIL-R2 were comparable in HDMSCs and ASMSCs. (a) GFP-positive cells were observed under a confocal microscope (40x), and the transduction efficiencies were comparable between HDMSCs and ASMSCs. (b) The lentiviruses encoding shRNA-TRAIL-R2 inhibited both the gene and protein expression of TRAIL-R2 in HDMSCs (*n* = 28) and ASMSCs (*n* = 22). Values are presented as the mean ± SD. *∗* indicates *P* < 0.05 between HDMSCs and ASMSCs. & indicates *P* < 0.05 for the TNF-*α*/CHX group compared with other HDMSCs groups. # indicates *P* < 0.05 for the TNF-*α*/CHX group compared with other ASMSCs groups. Lv-NC refers to MSCs transduced by lentiviruses encoding a negative control sequence. Lv-TRAIL-R2 refers to MSCs transduced by lentiviruses encoding TRAIL-R2.

**Figure 6:**
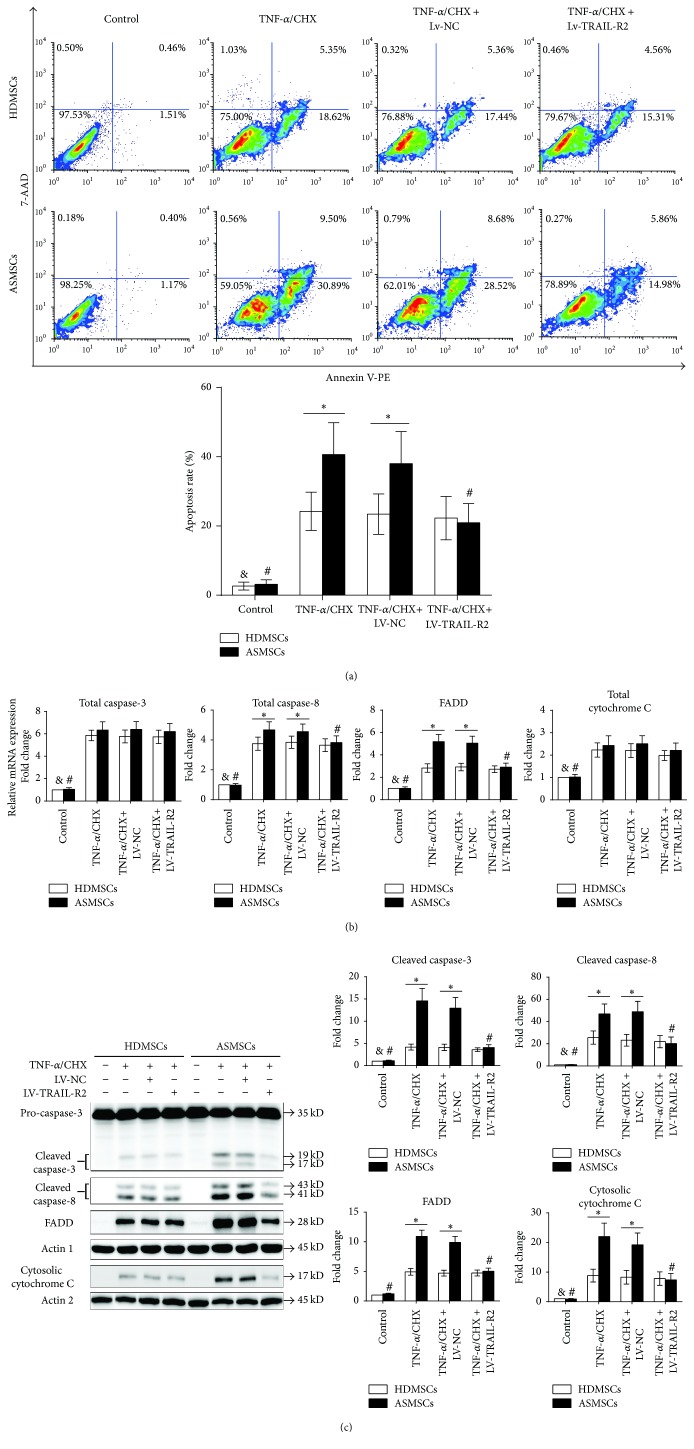
Lentiviruses encoding shRNA-TRAIL-R2 partly inhibited the apoptosis of ASMSCs while having a limited effect on the apoptosis of HDMSCs. (a) The rate of ASMSC apoptosis (*n* = 22) was decreased after Lv-TRAIL-R2 transduction, while no apparent changes were found for HDMSCs (*n* = 28). (b) In ASMSCs (*n* = 22), Lv-TRAIL-R2 reduced FADD and total caspase-8 expression while having a limited effect on total caspase-3 and total cytochrome C gene expression. In HDMSCs (*n* = 28), Lv-TRAIL-R2 had a limited effect on the expression of all four genes. (c) Lv-TRAIL-R2 did not affect the protein levels of cleaved caspase-3, cleaved caspase-8, FADD, or cytosolic cytochrome C in HDMSCs (*n* = 28) but reduced them in ASMSCs (*n* = 22). Values are presented as the mean ± SD. *∗* indicates *P* < 0.05 between HDMSCs and ASMSCs. & indicates *P* < 0.05 for the TNF-*α*/CHX group compared with other HDMSC groups. # indicates *P* < 0.05 for the TNF-*α*/CHX group compared with other ASMSC groups. Lv-NC refers to MSCs transduced by lentiviruses encoding a negative control sequence. Lv-TRAIL-R2 refers to MSCs transduced by lentiviruses encoding TRAIL-R2.

Because ASMSCs express more TRAIL-R2 than HDMSCs during apoptosis, we constructed Lv-TRAIL-R2 to explore the role that TRAIL-R2 plays in the apoptosis of ASMSCs. GFP-positive cells were observed under a confocal microscope, and the transduction efficiencies were comparable between HDMSCs and ASMSCs (Figure 5(a)). Lv-TRAIL-R2 inhibited both gene and protein expression of TRAIL-R2 in HDMSCs and ASMSCs (Figure 5(b)). The FCM results demonstrated that Lv-TRAIL-R2 could reduce the apoptosis of ASMSCs but had a limited effect on the apoptosis of HDMSCs (Figure 6(a)). qRT-PCR showed that Lv-TRAIL-R2 inhibited the expression of FADD and total caspase-8 in ASMSCs but had a limited effect on total caspase-3 and total cytochrome C. Meanwhile, Lv-TRAIL-R2 did not affect the expression of the four related aforementioned genes in HDMSCs. After inhibiting TRAIL-R2 expression, no apparent difference in gene expression was found between HDMSCs and ASMSCs (Figure 6(b)). Additionally, Lv-TRAIL-R2 did not affect the protein levels of cleaved caspase-3, cleaved caspase-8, FADD, or cytosolic cytochrome C in HDMSCs but reduced them in ASMSCs. Furthermore, no apparent difference in the protein expression of cleaved caspase-3, cleaved caspase-8, FADD, or cytosolic cytochrome C was found between HDMSCs and ASMSCs after inhibiting TRAIL-R2 expression (Figure 6(c)). Therefore, inhibiting TRAIL-R2 expression partly decreased the apoptosis of ASMSCs but had a limited effect on HDMSCs.

## 4. Discussion

Abnormal MSCs are proposed to be involved in the pathogenesis of AS, and a better understanding of ASMSCs dysfunctions could improve the understanding of AS. In this study, ASMSCs had morphologies, phenotypes, and proliferation rates that were similar to HDMSCs. However, ASMSCs were more susceptible to TNF-*α*/CHX-induced apoptosis compared to HDMSCs. ASMSCs expressed higher levels of TRAIL-R2, which activated both the DR and the mitochondrial pathways by increasing the expression levels of FADD, cleaved caspase-8, and cytosolic cytochrome C. Inhibiting TRAIL-R2 expression using shRNA eliminated the difference in apoptosis between HDMSCs and ASMSCs by decreasing the apoptosis of ASMSCs.

MSCs are multipotent stromal cells with multilineage differentiation capacities and immunomodulatory properties. Our previous studies suggested that MSCs are implicated in the pathogenesis and therapy of AS [12, 28]. We found that ASMSCs possessed an abnormal osteogenic capacity, which may contribute to the pathogenesis of AS. In addition, we also found that allogenic intravenous infusion of bone marrow-derived MSCs is feasible and safe in the treatment of AS. However, how the in vivo microenvironment in AS interacts with MSCs remains unknown.

AS is a complicated autoimmune disease accompanied by elevated serum levels of various inflammatory cytokines [14, 29]. Among these cytokines, TNF-*α* is one of the most important players in the pathogenesis of AS. Physiologically, TNF-*α* is important for a normal response to inflammation, but the overproduction of TNF-*α* can be detrimental. Serum levels and the sacroiliac joint expression of TNF-*α* were elevated in AS patients, including those in the early stages of AS [30, 31]. In addition, overproduction of TNF-*α* has been suggested to be related to disease activity and to account for the pathogenesis of AS [32]. The importance of TNF-*α* has been further confirmed by the efficacy of TNF-*α* inhibitors in the treatment of AS [16]. Due to the importance of TNF-*α* in AS, a study on the interaction between TNF-*α* and MSCs may help mimic the in vivo microenvironment experienced by MSCs in AS. Previous studies suggest that TNF-*α* affects MSCs in various ways. TNF-*α* can modulate the immunosuppressive effects of MSCs and suppress their osteogenic capacity [33]. Additionally, TNF-*α* can alter homeostasis in MSCs by regulating the balance between apoptosis and survival [13]. Conversely, MSCs affect the production of TNF-*α* by causing macrophages to adopt an anti-inflammatory phenotype [34]. Both the dysfunction of MSCs and the overproduction of TNF-*α* may lead to immune disorders. A recent study suggested that TNF-*α*-induced apoptosis of MSCs may contribute to the pathogenesis of SLE [19]. In our study, we found that ASMSCs were more susceptible to TNF-*α*-induced apoptosis compared to HDMSCs, as evidenced by an increased apoptosis rate and the higher expression of cleaved caspase-3 protein. However, how the dysregulation of apoptosis contributes to the pathogenesis of AS remains uncertain.

Apoptosis, also defined as programmed cell death, is critical to the pathogenesis of various diseases, including autoimmune diseases [18]. The maintenance of immune tolerance and immune homeostasis is largely dependent on apoptosis. Defective apoptosis of immune cells may result in the defective clearance of autoreactive cells or the delayed elimination of autoantigens, while excessive apoptosis of immunosuppressive cells (such as MSCs) may also lead to immune hyperactivation. Therefore, both conditions may contribute to autoimmune disease [19, 35]. In patients with AS, increased apoptosis of ASMSCs may result in decreases in cell numbers and immunosuppressive potential, eventually contributing to autoimmune disorders. Moreover, because MSCs are able to reduce TNF-*α* production, increased MSCs apoptosis may also lead to TNF-*α* overproduction. Furthermore, TNF-*α* overproduction induces further MSC apoptosis, forming a vicious cycle that leads to chronic inflammation in AS. Thus, increased ASMSC apoptosis may be critical for AS pathogenesis, and further study of the mechanism of dysregulated apoptosis in ASMSCs may help to elucidate the pathogenesis of AS.

TRAIL-R2 is a specific cell surface receptor engaged in apoptosis. TRAIL-R2-mediated apoptosis has aroused attention due to its role in preferentially killing tumor cells while sparing normal cells. In our study, we observed a significant increased expression of TRAIL-R2 in ASMSCs after treatment with TNF-*α*/CHX. Inhibiting TRAIL-R2 expression partly decreased the apoptosis of ASMSCs but had a limited effect on the apoptosis of HDMSCs, which suggests that TRAIL-R2 overexpression is the main cause of the increased apoptosis in ASMSCs. However, this result is confusing for two reasons. First, TRAIL-R2 is reported to be mainly activated by its specific ligand, TRAIL [20]. However, the results in our study indicated that TRAIL-R2 in MSCs could be activated by TNF-*α*/CHX as well. In fact, a similar result was previously reported, but the mechanism remains unclear [36]. Considering the overexpression of TRAIL-R2 can lead to apoptosis even without its ligand [22], we speculate that TNF-*α* may mediate its proapoptotic effects not only through its own receptor (TNFR1) but also by increasing the expression of TRAIL-R2. Second, bone marrow-derived adult MSCs are reportedly resistant to TRAIL-R2-mediated apoptosis [21]. However, in our study, HDMSCs were resistant to TRAIL-R2-mediated apoptosis, while ASMSCs were not. Multiple mechanisms of TRAIL-R2-mediated apoptosis resistance, such as dysregulation of antagonistic receptors, abnormal O-glycosylation, and overexpression of the inhibition adaptors, have been identified [37]. Further studies are needed to illustrate the associated specific mechanism underlying the difference in resistance of HDMSCs and ASMSCs to TRAIL-R2. Additionally, based on the finding that the TRAIL-R2 pathway did not lead to the apoptosis of normal cells, ASMSCs could be considered abnormal cells. Thus, it is reasonable to presume that allogenic rather than autologous MSC transplantation might be preferable for AS treatment.

In this study, we demonstrated that TRAIL-R2 upregulation resulted in enhanced apoptosis of ASMSCs. Increased apoptosis of ASMSCs may decrease the immunosuppressive potential of these cells and potentially contributing to AS pathogenesis. However, some limitations remain in this study. The questions of how TNF-*α* activates the expression of TRAIL-R2, why HDMSCs are resistant to TRAIL-R2-mediated apoptosis while ASMSCs are not, and whether our results can be repeated in vivo remain unanswered. Future studies should focus on these questions and further elucidate the mechanism of AS.

## Supplementary Material

Base line characteristics of the participants.
