# Supplementary material for: TNF-α Induced the Enhanced Apoptosis of Mesenchymal Stem Cells in Ankylosing Spondylitis by Overexpressing TRAIL-R2
Source: Stem Cells Int. 2017 Jan 15;2017:4521324. doi: 10.1155/2017/4521324 (PMC5274669; doi:10.1155/2017/4521324)
Supplement: Supplementary file 1 — Base line characteristics of the participants. [file 4521324.f1.pdf]

Supplement Table 1. Characteristics of the study subjects

	**Healthy donors**	**AS patients**
Number	28	22
Age (year)	30.2 ±10.1	34.8 ±12.8
Male no.(%)	18（64.3%）	15（68.2%）
HLA-B27 positive no.(%)	0	21（95.5%）*
Disease duration (year)	NA	7.02 ±6.2
CRP (mg/L)	3.1 ±1.3	18.6 ±13.2*
ESR (mm/h)	10.1 ±3.3	27.5 ±14.9*
BASDAI	0.95 ±0.86	4.69 ±1.01*
Treatment options		
NSAIDs	0	10
TNF-α inhibitor	0	7
NSAIDs+DMARDs	0	5

Mean ±SD. AS, ankylosing spondylitis; HLA-B27, human leukocyte antigen B27; CRP, C-reactive protein; ESR, erythrocyte sedimentation rate; BASDAI, the bath ankylosing spondylitis disease activity index; * indicates P< 0.05 compared to healthy donors.
